# Chirped guided-mode resonance biosensor

**DOI:** 10.1364/OPTICA.4.000229

**Published:** 2017-02-13

**Authors:** Graham J. Triggs, Yue Wang, Christopher P. Reardon, Matthias Fischer, Gareth J. O. Evans, Thomas F. Krauss

**Affiliations:** 1 Department of Physics, University of York, York YO10 5DD, UK; 2 Department of Biology, University of York, York YO10 5DD, UK

**Keywords:** (050.2770) Gratings, (050.5745) Resonance domain, (050.6624) Subwavelength structures, (280.1415) Biological sensing and sensors, (280.4788) Optical sensing and sensors

## Abstract

Advanced biomedical diagnostic technologies fulfill an important role in improving health and well-being in society. A large number of excellent technologies have already been introduced and have given rise to the “lab-on-a-chip” paradigm. Most of these technologies, however, require additional instrumentation for interfacing and readout, so they are often confined to the laboratory and are not suitable for use in the field or in wider clinical practice. Other technologies require a light coupling element, such as a grating coupler or a fiber coupler, which complicates packaging. Here, we introduce a novel biosensor based on a chirped guided-mode resonant grating. The chirped grating combines the sensing function with the readout function by translating spectral information into spatial information that is easily read out with a simple CMOS camera. We demonstrate a refractive index sensitivity of 137 nm/RIU and an extrapolated limit of detection of 267 pM for the specific binding of an immunoglobulin G antibody. The chirped guided-mode resonance approach introduces a new degree of freedom for sensing biomedical information that combines high sensitivity with autonomous operation. We estimate that the cost of components is U.S. $10 or less when mass manufactured, so the technology has the potential to truly transform point-of-care applications.

## 1. INTRODUCTION

Biosensors present one of the most promising opportunities for the silicon photonics paradigm, as they combine high sensitivity with mass-manufacturing capability. For example, the Genalyte Maverick system [1] is based on microring resonators [2] that are manufactured in a CMOS foundry and that provide some of the highest biomarker detection sensitivities available. The microring is one of a family of resonant photonic biosensors that detect biomarkers via a change in the refractive index upon molecular binding, alongside photonic crystals [3], resonant gratings [4], surface plasmon sensors [5] and fiber Bragg gratings [6]. A key advantage of such biosensors is that they afford highly sensitive and real-time detection without the need for a tag, label, or reporter molecule, i.e., they are label free. Conventionally, they achieve their high performance by tracking a narrow photonic resonance via a tunable light source or a high-resolution spectrometer, and the desired biomedical information is encoded spectrally as a wavelength shift. A key issue, therefore, is that readout tools add significantly to the size, complexity, and cost of the sensor system and confine its use to a laboratory with trained users: the so-called “lab-on-a-chip.” Another issue is the need to interface the light source with the sensor, e.g., via a grating coupler or an end-fire coupler. In waveguide-based devices, a coupler is needed to excite the waveguide mode, which undergoes resonance or is fed into an interferometric structure, such as a Mach–Zehnder interferometer. Interferometric sensors, in particular, can achieve sensitivities of up to $10^{-8}$ refractive index units [7]. In order to make photonic biosensors more widely applicable, the challenge is to translate the technology into a low-cost, small-footprint, stand-alone device that is robust and that can be used in the field by untrained personnel.

A number of solutions have already been developed to address this challenge and to push resonant biosensors toward a true lab-on-a-chip technology, with many of them aiming to directly interface the biosensor with a smartphone [8–12]. The issue of providing an integrated readout in such a smartphone biosensor has been addressed by employing external gratings or prisms to disperse the spectral information onto the camera sensor. This solution, while elegantly replacing the external spectrometer, is often alignment sensitive and carries the risk of shock sensitivity when devices are used in the field, as well as requiring long optical paths to achieve the desired spectral resolution. Therefore, the use of external gratings or prisms tends to result in bulky attachments [9]. Furthermore, while it is clearly attractive to design the sensor as a smartphone attachment that uses the in-built camera and light source, the need for bespoke cradles for each smartphone model on the market quickly outstrips the cost advantage, especially since high-performance CMOS cameras can be acquired for a few USD and suitable light sources for even less. In addition, the alignment of the cradle to the source and camera is often critical, which increases the risk of user error.

The solution we present here utilizes a novel chirped resonant grating. The chirped grating not only generates the resonance signal, but it also translates the spectral information into spatial information using the same chip, thus elegantly combining the sensing function and enabling readout with simple optical elements and a camera. Both chirped grating couplers [13,14] and guided-mode resonances [15] for sensing applications have already been demonstrated, but their combination in a single structure is unique. Our sensor can be assembled in a similar fashion to the optical elements of a CD-ROM drive, so it is mass manufacturable, rugged, low cost, and requires no user alignment or adjustment. The device may also be encapsulated and equipped with a battery and micro-PC to allow operation in aqueous environments, e.g., for the monitoring of biofouling [16]. Importantly, these advantages do not have to be traded off against low performance. We demonstrate this with a sensitivity of 137 nm/RIU, a refractive index limit of detection of $2.37\times 10^{-4}$ RIU, and an extrapolated limit of detection of 40 ng/mL (267 pM) for the specific binding of an immunoglobulin G (IgG) antibody. This performance lies in the clinically relevant concentration regime; for example, the concentration of human allergy antibody IgE in blood serum from patients sensitized to allergic diseases and parasitic infections is reported to be around 300 ng/mL [17].

## 2. MATERIALS AND METHODS

### A. Chirped Grating Design and Fabrication

The key element of our biosensor is the chirped resonant grating. This is fabricated in a 150 nm thick silicon nitride (${\mathrm{Si}}_{3}N_{4}$) film on glass using electron beam lithography and reactive ion etching. We note that e-beam lithography could be employed to fabricate thousands of individual grating elements from a single wafer, which would keep the production cost very low, especially considering 1  mm×1  mm gratings can be now be exposed in as little as ∼5  min [18]. Nanoimprint lithography could also be used for large-scale production at even lower costs, less than U.S. $1 per grating. The motivation for choosing ${\mathrm{Si}}_{3}N_{4}$ is its high refractive index and minimal absorption at visible and near-infrared wavelengths [19,20], its biocompatibility [21], chemical inertness [22], and its excellent mechanical robustness, which allows for multiple re-uses after cleaning. The grating dimensions are based on those reported in our previous work [23]: a period (a) of 560 nm and a filling factor (or FF: the percentage of the period that is taken by the grating ridge, i.e., the fraction of high-refractive index material) of 0.7, which were determined using rigorous coupled-wave analysis (RCWA) simulations [24,25]. First, the ${\mathrm{Si}}_{3}N_{4}$ is cleaned in a piranha solution (1:3 hydrogen peroxide: sulfuric acid), rinsed in acetone and isopropanol, and dried with nitrogen. Next, the e-beam resist (ARP-09, AllResist GmbH) is spin-coated for 60 s at 3000 rpm, and soft baked on a hot plate at 180°C for 10 min. For charge dissipation during e-beam exposure, a thin film of aluminum (∼20  nm) is deposited on top of the resist using a thermal evaporator (HEX, Mantis). The e-beam system (Voyager, Raith GmbH) exposes the resist with a base dose of $130\;\mathrm{\mu C}/{\mathrm{cm}}^{2}$, after which it is developed for 2 min in Xylene, then rinsed with isopropanol and dried in $N_{2}$. To transfer the grating into the ${\mathrm{Si}}_{3}N_{4}$, it is etched with a ${\mathrm{CHF}}_{3}\text{:}O_{2}$ gas mixture at 29:1 sccm, an RF power of 40 W, and a chamber pressure of $1.9\;e^{-1}$ mbar, over 7 min etch time. Finally, to strip the remaining resist, the sample is sonicated in 1165 solvent (MicroChem) for 3 min, followed by a rinse in acetone and isopropanol. It is dried with $N_{2}$.

**Fig. 1.**
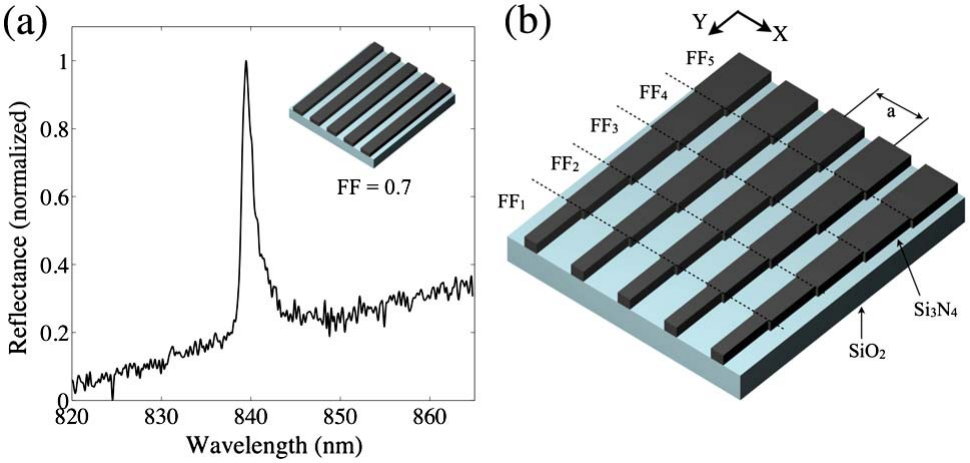
(a) Typical measured reflectance spectrum from a regular ${\mathrm{Si}}_{3}N_{4}$ grating. (b) The chirped grating design: the grating is composed of strips, each with a different FF, in order to chirp $\lambda_{R}$ as a function of its position along Y (FF change is exaggerated here for illustration purposes).

When the grating is illuminated with polarized, collimated light, a guided-mode resonance [26] is excited, resulting in a narrow-band reflectance peak at a resonance wavelength ($\lambda_{R}$) of around 840 nm [Fig. 1(a)], with a typical linewidth of ∼2  nm. The resonance emerges when light couples into a guided mode in the high-index ${\mathrm{Si}}_{3}N_{4}$ layer and is coherently backreflected from the grating interfaces. This forms a standing wave in the grating that has a significant overlap with the region outside the grating, thereby responding sensitively to changes in the external refractive index. The value of $\lambda_{R}$ hence provides an accurate indicator of the external refractive index and, via suitable biochemical functionalization, the concentration of a target biomarker. Usually, to monitor $\lambda_{R}$, a spectrometer or dispersive system is used. Here, however, in order to remove the need for such readout instruments, we obtain the spectral information by chirping the grating, thereby making $\lambda_{R}$ a function of the *position*, as described next.

**Fig. 2.**
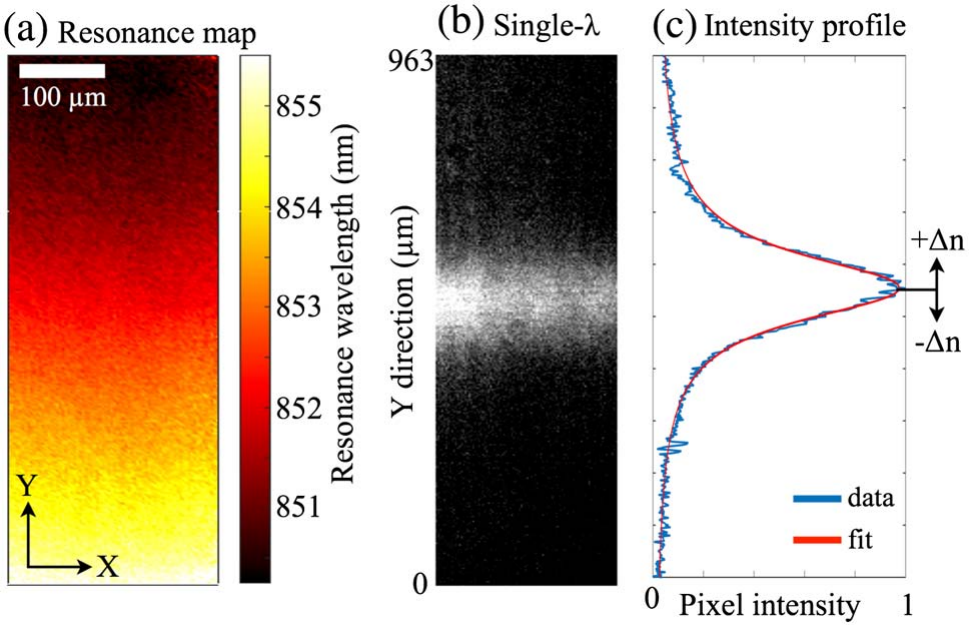
(a) Resonance wavelength map of the grating (water is the surrounding liquid). (b) Brightfield image of the grating under monochromatic illumination. (c) Intensity profile of the resonance shown in (b), averaged horizontally and normalized. The blue curve shows raw data, while the red curve shows a fitted Fano curve, as described in the text. The position of the resonance is monitored continually, allowing changes in the refractive index (Δn) to be detected.

Since $\lambda_{R}$ depends both on the grating period and the FF, tuning either one of these parameters will produce a chirp. Tuning the period is the most obvious approach, yet it is limited by the finite step size of an electron-beam lithography system (1 nm for our system), and this is too coarse a tuning method for the desired application. Instead, we gently alter the FF of the grating by changing the electron exposure dose during lithography, which affords a much finer tuning of $\lambda_{R}$. We note that the tuning range available via the FF is much smaller than via the period, so we set the center wavelength of the grating with the period and then vary the FF to fine-tune the wavelength, thus producing the chirp. The sensor is composed of individual grating strips, each with a different FF, as shown in Fig. 1(b). Specifically, the exposure dose of each strip ranges from 117 to $130\;\mathrm{\mu C}/{\mathrm{cm}}^{2}$. The grating strips are stacked in the Y-direction, i.e., perpendicular to the grating vector, which is oriented in the X-direction. This arrangement affords tighter stacking and less crosstalk between subsequent gratings because the resonant mode oscillates in the X-direction only, not in Y [23]. Here, each strip has a height of 6 μm (Y) and a width of 500 μm (X), but they could be made significantly smaller if further miniaturization is desired. To demonstrate the spatial tuning of $\lambda_{R}$, Fig. 2(a) shows a resonance wavelength map of the grating, obtained by sweeping the illumination wavelength while collecting brightfield images. The resulting stack of images is then processed to identify $\lambda_{R}$ for every pixel, and these values make up the map shown. It is clearly seen that $\lambda_{R}$ varies smoothly across the grating in the Y-direction. From this map, we observe a total shift in $\lambda_{R}$ of ∼5.5  nm. Using the RCWA simulations, we estimate the corresponding change in the FF is approximately 6%.

When the chirped grating is illuminated with monochromatic light, only a narrow horizontal strip resonates, causing high reflectance solely from this region, as shown in Fig. 2(b). The precise position of the resonance can be extracted by first averaging in the horizontal direction (X) and then fitting a curve to this averaged intensity profile [Fig. 2(c)]. We choose a Fano curve [27] because the physical origin of the resonance is the interplay between the Fabry–Perot resonance of the thin film and the Bragg resonance of the grating, although the Bragg resonance tends to dominate such that the Fano tends toward a Lorentzian lineshape, as is the case here, although the lineshape is still slightly asymmetrical. The fitting and determination of the peak location here are implemented using MatLab, utilizing a nonlinear-least-squares method. If there is a change in the refractive index (Δn) at the grating, the resonance *position* shifts accordingly, thus allowing binding events or refractive index changes to be quantified in real time, using only a basic camera as the readout instrument and a simple monochromatic light source.

### B. Measurement Setup and Microfluidics

Although the sensor could be packaged as a stand-alone device, here we demonstrate the proof-of-concept operation by measuring the resonance position using a simple inverted microscope setup at a low magnification. The camera employed here is a CoolSnap Myo (Photometrics), and the objective lens is an Olympus NeoDplan 5× (NA=0.13). The camera pixel size is 4.54 μm, and after magnification, each image pixel images a size of 1.85 μm. To provide some flexibility in matching the illumination wavelength to the $\lambda_{R}$ of the fabricated gratings, we use a broadband laser (SM30, Leukos), combined with a custom-built grating-based monochromator to select a single illumination wavelength with a spectral width of 0.6 nm. Images were captured using LabView, and image analysis and curve fitting were performed using MatLab.

We also developed a microfluidic channel made from a molded polydimethylsiloxane (PDMS) elastomer, which is attached to the sensor chip to allow exposure to various analytes. The mold was created with SU-8 2050 resist (MicroChem), diluted using cyclopentanone to give a thickness of ∼100  μm. This is spin-coated onto a silicon wafer and soft baked at 95°C for 20 min before being exposed using an e-beam with a dose of $5\;\mathrm{\mu C}/{\mathrm{cm}}^{2}$. After exposure, it is baked again at 95°C for 20 min, developed in EC-solvent (Shipley) for 2 min, and rinsed in isopropanol. The mold is hard baked at 160°C overnight; then, the PDMS elastomer (poly-dimethylsiloxane, Dow Corning) is poured on top and allowed to cure overnight at 60°C. The PDMS is used at a ratio of 10:1 elastomer:hardener. After curing, holes are punched through the PDMS, and it is then fixed onto the sensor chip using uncured PDMS as a glue. This is cured at 60°C for 3 h, and finally, Tygon tubing is inserted into the holes. A basic syringe pump was used here to pull the analyte through the channels and across the sensor chip. Importantly, since we observe the signal in reflection, the region above the grating is completely unrestricted and may be interfaced with any type of flow channel or fluid delivery system, such as paper, or it could be left exposed to the environment if desired for a given application.

## 3. RESULTS

### A. Sensitivity Measurements Using Glucose Solutions

**Fig. 3.**
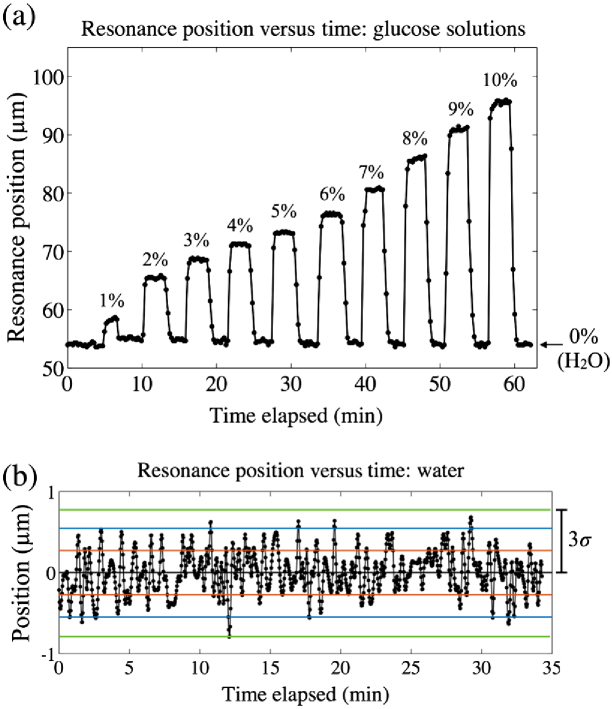
(a) Position of the resonance peak versus time as different glucose solutions are introduced, where the percentages indicate the %w/v glucose concentration. Water (0%) was introduced between each sugar solution. (b) Position versus time for water only, centered about the mean. The red, blue, and green lines indicate one, two, and three standard deviations (σ) away from the mean, respectively.

The sensitivity to refractive index changes is a key figure of merit for resonant photonic biosensor performance. Sensitivity is typically reported in units of nm/refractive index unit (RIU), a measure of how far the resonance wavelength, $\lambda_{R}$, shifts for a unit change of the refractive index. The performance of our sensor was determined by flowing a series of glucose solutions through the microfluidic channel while recording the position of the resonant strip [see Fig. 3]. By changing the concentration of dissolved glucose in deionized water, the refractive index can be controlled accurately. Here, the sensor is exposed to glucose concentrations from 0% to 10% w/v in steps of 1%. This corresponds to a refractive index range of 1.3324 to 1.3444 in steps of $1.2\times 10^{-3}$ RIU [28].

The average positional shift per RIU is measured to be 3469 μm/RIU. This value should not be compared to a conventional nm/RIU sensitivity, since we measure a position instead of a resonance wavelength; indeed, $\lambda_{R}$ remains fixed, so the usual figure of merit is inappropriate. Nevertheless, we have measured the wavelength shift from a nonchirped resonant grating of exactly the same structure, also in response to glucose solutions (see Fig. S1 in Supplement 1). For this case, we find a sensitivity of 137 nm/RIU, which is in agreement with other values for resonant grating sensors reported in the literature [29]. We estimate the smallest detectable shift to be 3 times the standard deviation (σ) in position for 0% glucose (water). This is measured over ∼35  min to be 0.82 μm [Fig. 3(b)]. Dividing this by the average positional sensitivity (3469 μm/RIU) gives a limit of detection of $2.37\times 10^{-4}$ RIU, in excellent agreement with other photonic crystal biosensors [30]. This value is already sufficient for detecting clinically relevant concentrations of biomolecules, as we show next, but could be further improved toward values of $10^{-5}$ by using an even finer dose variation across the grating or a narrower-bandwidth light source.

### B. Detection of IgG Binding

**Fig. 4.**
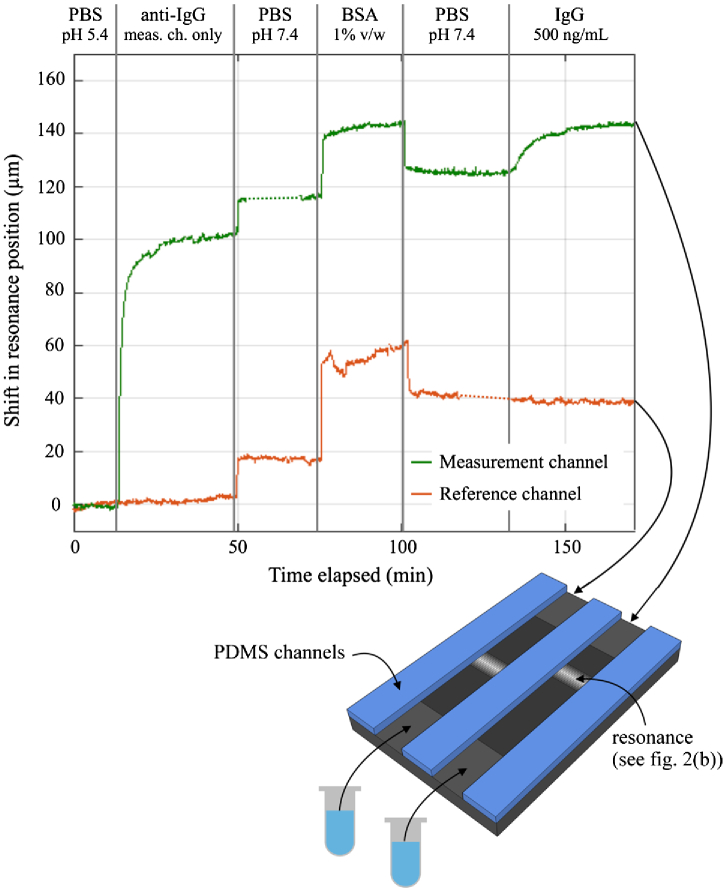
IgG binding assay showing the shift in resonance position against time for both the measurement and reference channels, with each assay step indicated. The two small dashed regions on the curves correspond to where the signal was lost momentarily due to the flow channel becoming partially blocked. The illustration underneath shows the flow channel configuration used for the experiment: two separate channels allow one to be functionalized and the other kept as a reference. The analyte was pulled through the channels by a single syringe pump operating with two separate syringes, one for each channel.

To study the biomolecular detection capability of our biosensor, we measure an antigen (IgG) binding to its corresponding antibody (anti-IgG). First, the sensor is cleaned in piranha (see the Methods section) and immediately placed overnight in a 5% solution of APTES:ethanol. APTES is 3-aminopropyl-triethoxysilane (Sigma), and this salinization treatment creates free amine groups on the surface to which antibodies can bind. After this stage, the flow channel is bonded to the chip, as described previously. We note here that silane layers are stable in the 60°C curing temperature used to bond the PDMS channel to the chip, as they are reported to withstand curing temperatures of up to 250°C [31]. In particular, to allow a reference measurement to be done simultaneously, we use two separate channels positioned over adjacent regions of the grating (see Fig. 4). To render the sensor specific to IgG, *one* channel is functionalized with anti-IgG, immobilized using the NHS-EDC protocol [32], whereby a covalent bond is formed between the carboxyl group on the antibody and the amine groups on the prepared sensor. Specifically, NHS (N-Hydroxysulfosuccinimide sodium salt, 56485, Sigma) and EDC (N-(3-Dimethylaminopropyl)-N-ethylcarbodiimide hydrochloride, 03449, Sigma) are dissolved in phosphate-buffered saline (PBS) at pH 5.4 to concentrations of 10 and 8 mg/mL, respectively. These are added to the antibody (Anti-Rabbit IgG, R2004, Sigma), which is dissolved in deionized water. The final antibody concentration is 50 μg/mL. This mixture is reacted for 20 min before being introduced to the chip.

Figure 4 shows the shift in the resonance position against time as the IgG binding assay proceeds for both the measurement channel (functionalized with anti-IgG) and the reference channel. The assay stages are as follows. After establishing a baseline with PBS at pH 5.4, there is a clear shift during antibody binding in the measurement channel (the reference channel is not exposed to the antibody solution). The anti-IgG binding curve saturates after ∼30  min as the binding sites become used up. Antibody binding is followed with a PBS wash at pH 7.4, which induces a step change in both channels due to a higher bulk refractive index of the solutions. Next, we introduce a blocking buffer (bovine serum albumin (BSA), diluted to 1% w/v in PBS), then a further wash in PBS. There is a resonance shift caused by BSA binding in both channels, showing that the nonspecific binding sites are being occupied by the BSA. Moreover, the shift is clearly smaller in the measurement channel, due to the antibody coverage. There are fewer nonspecific sites here for the BSA to bind to. Finally, we introduce the IgG antigen (I5006, Sigma) into both channels, at a concentration of 500 ng/mL, diluted in PBS. The IgG is detected after only 2–3 min, the short timescale being highly valuable in point-of-care applications. The lack of shift in the reference channel clearly indicates the site-specific binding of IgG antibody in the measurement channel.

As before, an estimation of the detection limit can be made. Three times the standard deviation (3σ) of a flat region of the curve (the initial 10 min of PBS at pH 5.4) is measured to be 1.458 μm. (N.B. This value is larger than that shown previously in Fig. 3(b) because it is a different grating design, using strips that are approximately twice as tall as before, and thus, the resonance is broader.) Since the 500 ng/mL concentration causes a shift of 18 μm, and assuming a linear response, the smallest detectable concentration is approximately 40 ng/mL. This corresponds to a molar concentration of approximately 267 pM for IgG, assuming a molecular weight of 150 kDa.

## 4. DISCUSSION

Having now introduced the chirped grating modality as means to facilitate a simple biosensor readout, one can consider further variations, such as introducing a chirp in two dimensions to add further degrees of freedom without adding any complexity, since the detector CCD is already a two-dimensional array. Also, as already demonstrated above for two channels, multiple flow channels can be monitored simultaneously on a single chip. This opens up possibilities for the detection of multiple biomarkers in parallel from a single sample. A related step in this direction would be to harness the scalability of e-beam lithography and CMOS processing. Thousands of chirped grating elements could be mass produced from a single wafer of material, drastically lowering production costs. Together with a simple configuration reminiscent of the optical elements of a CD-ROM drive, we estimate that the entire sensor could be mass manufactured for less than U.S. $10. Due to the configuration of the system, where the resonance position is self-referencing via the grating boundaries or dedicated alignment markers, no active alignment is required, and the grating chip can be easily replaced without the need for careful optical alignment. This also makes the sensor very robust to mechanical shocks and thermal drifting, since systematic movements in the position of the chip can easily be identified and cancelled out.

Additionally, because the spectral information is encoded spatially on-chip and the system works at the single wavelength given by the light source, the spectral response of the camera does not come into play. Compared to readout systems where dispersion is used as the transduction method, we remove one further source of error. By operating at a single wavelength, we can also choose the optimum wavelength to suit the camera, thus achieving the best signal-to-noise ratio. Also, since the sensing information is encoded as a relative shift in the pixel position, the measurement is intrinsically self-referencing and therefore more accurate than that obtained by recording intensity changes alone.

Another possibility arises by forming a sensor array from small, individual chirped gratings, thereby making it possible to *image* local variations in refractive index shifts at a video rate. This would be a powerful tool for monitoring inhomogeneous substances, such as biofilm growth, or for high-throughput drug screening by simultaneously probing many regions. Such a sensing-imaging modality has already been demonstrated [23,33], yet only with systems that require either the sweeping of the incidence wavelength or the angle, so complex setups and relatively long acquisition times are needed.

## 5. CONCLUSIONS

We have demonstrated a unique realization of a resonant photonic biosensor that uses a novel chirped grating element. Our approach works by encoding spectral resonance information into spatial information using a single chip, thus removing any requirement for a spectrometer or dispersive optics. Readout is achieved simply by imaging the chip onto a CMOS camera and does not require a bespoke setup or a smartphone attachment. Despite its inherent simplicity, our biosensor does not compromise on performance. The sensitivity of 137 nm/RIU, LOD of $2.37\times 10^{-4}$ RIU, and the ability to detect a 40 ng/mL (267 pM) concentration of a target molecule within a few minutes is already sufficient for many practical applications. By packaging the chip with basic optical elements and a camera, the whole device could be made portable, stand-alone, compact, and highly robust, as well as easy to use and inexpensive to manufacture. The reflectance configuration lends itself to interfacing with almost any type of analyte delivery method, and it could be remotely operated. Furthermore, there is still plenty of room for improvement, such as using a narrow-bandwidth light source, optimizing the integration time, and other engineering issues, so we believe the chirped grating biosensor will come to play an important role as a diagnostics tool for clinical, home, and many other biosensing applications.

## Notes

See Supplement 1 for supporting content.
