# Supplementary material for: Chirped guided-mode resonance biosensor
Source: Optica. 2017 Feb 13;4(2):229–34. doi: 10.1364/OPTICA.4.000229 (PMC6513287; doi:10.1364/OPTICA.4.000229)
Supplement: Supplementary file 1 [file optica-4-2-229-s001.PDF]

optica

Chirped guided-mode resonance biosensor: supplementary material

Graham J. Triggs[1], Yue Wang[1,*], Christopher P. Reardon[1], Matthias Fischer[1], Gareth J. O. Evans[2], and Thomas F. Krauss[1]

[1] *Department of Physics, University of York, York, UK, YO10 5DD*
[2] *Department of Biology, University of York, York, UK, YO10 5DD*
[*] *Corresponding author: yue.wang@york.ac.uk*

Published 13 February 2017

This document provides supplementary information to "Chirped guided-mode resonance biosensor," https://doi.org/10.1364/optica.4.000229. © 2017 Optical Society of America

https://doi.org/10.1364/optica.4.000229.s001

The sensitivity of the gratings employed for our chirped grating sensor has been measured using the same glucose solutions as reported in the main paper, where the resonance wavelength (λ_R) of a standard non-chirped grating was monitored versus time (figure S1 below). Here, instead of a spatial measurement of the resonance shift, we directly measure the reflectance spectrum using a spectrometer (Thorlabs CCS175). The measured data also underwent a Fano curve fitting, as before, from which λ_R is extracted. From the average resonance shift at each step, and the knowledge of the refractive index of the glucose solutions, we obtain a sensitivity of 137 nm/RIU $\pm$ 3 nm/RIU.

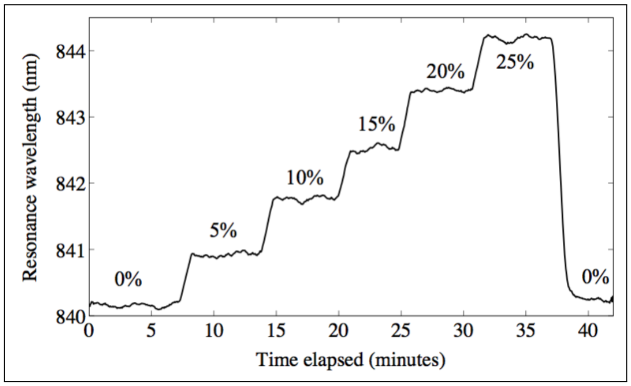

Fig. S1. Figure showing resonance wavelength (nm) versus time as glucose solutions are introduced to a standard non-chirped grating as discussed above.
